# Maternal age and severe maternal morbidity: A population-based retrospective cohort study

**DOI:** 10.1371/journal.pmed.1002307

**Published:** 2017-05-30

**Authors:** Sarka Lisonkova, Jayson Potts, Giulia M. Muraca, Neda Razaz, Yasser Sabr, Wee-Shian Chan, Michael S. Kramer

**Affiliations:** 1 Department of Obstetrics and Gynaecology, University of British Columbia and Children’s and Women’s Health Centre of British Columbia, Vancouver, British Columbia, Canada; 2 School of Population and Public Health, University of British Columbia, Vancouver, British Columbia, Canada; 3 Department of Medicine, University of British Columbia and BC Women’s Hospital and Health Centre, Vancouver, British Columbia, Canada; 4 Clinical Epidemiology Unit, Department of Medicine, Karolinska University Hospital, Karolinska Institutet, Stockholm, Sweden; 5 College of Medicine, King Saud University, Riyadh, Saudi Arabia; 6 Department of Pediatrics, Faculty of Medicine, McGill University, Montreal, Quebec, Canada; 7 Department of Epidemiology, Biostatistics and Occupational Health, Faculty of Medicine, McGill University, Montreal, Quebec, Canada; University of Manchester, UNITED KINGDOM

## Abstract

**Background:**

One of the United Nations’ Millennium Development Goals of 2000 was to reduce maternal mortality by 75% in 15 y; however, this challenge was not met by many industrialized countries. As average maternal age continues to rise in these countries, associated potentially life-threatening severe maternal morbidity has been understudied. Our primary objective was to examine the associations between maternal age and severe maternal morbidities. The secondary objective was to compare these associations with those for adverse fetal/infant outcomes.

**Methods and findings:**

This was a population-based retrospective cohort study, including all singleton births to women residing in Washington State, US, 1 January 2003–31 December 2013 (*n = * 828,269).

We compared age-specific rates of maternal mortality/severe morbidity (e.g., obstetric shock) and adverse fetal/infant outcomes (e.g., perinatal death). Logistic regression was used to adjust for parity, body mass index, assisted conception, and other potential confounders. We compared crude odds ratios (ORs) and adjusted ORs (AORs) and risk differences and their 95% CIs.

Severe maternal morbidity was significantly higher among teenage mothers than among those 25–29 y (crude OR = 1.5, 95% CI 1.5–1.6) and increased exponentially with maternal age over 39 y, from OR = 1.2 (95% CI 1.2–1.3) among women aged 35–39 y to OR = 5.4 (95% CI 2.4–12.5) among women aged ≥50 y. The elevated risk of severe morbidity among teen mothers disappeared after adjustment for confounders, except for maternal sepsis (AOR = 1.2, 95% CI 1.1–1.4). Adjusted rates of severe morbidity remained increased among mothers ≥35 y, namely, the rates of amniotic fluid embolism (AOR = 8.0, 95% CI 2.7–23.7) and obstetric shock (AOR = 2.9, 95% CI 1.3–6.6) among mothers ≥40 y, and renal failure (AOR = 15.9, 95% CI 4.8–52.0), complications of obstetric interventions (AOR = 4.7, 95% CI 2.3–9.5), and intensive care unit (ICU) admission (AOR = 4.8, 95% CI 2.0–11.9) among those 45–49 y. The adjusted risk difference in severe maternal morbidity compared to mothers 25–29 y was 0.9% (95% CI 0.7%–1.2%) for mothers 40–44 y, 1.6% (95% CI 0.7%–2.8%) for mothers 45–49 y, and 6.4% for mothers ≥50 y (95% CI 1.7%–18.2%). Similar associations were observed for fetal and infant outcomes; neonatal mortality was elevated in teen mothers (AOR = 1.5, 95% CI 1.2–1.7), while mothers over 29 y had higher risk of stillbirth. The rate of severe maternal morbidity among women over 49 y was higher than the rate of mortality/serious morbidity of their offspring. Despite the large sample size, statistical power was insufficient to examine the association between maternal age and maternal death or very rare severe morbidities.

**Conclusions:**

Maternal age-specific incidence of severe morbidity varied by outcome. Older women (≥40 y) had significantly elevated rates of some of the most severe, potentially life-threatening morbidities, including renal failure, shock, acute cardiac morbidity, serious complications of obstetric interventions, and ICU admission. These results should improve counselling to women who contemplate delaying childbirth until their forties and provide useful information to their health care providers. This information is also useful for preventive strategies to lower maternal mortality and severe maternal morbidity in developed countries.

## Abstract 2

Using population-based data including all singleton births to women residing in Washington State 2003 to 2013, Sarka Lisonkova and colleagues calculated age-specific rates of adverse maternal and neonate outcomes.

## Author summary

**Why was this study done?:**

- Average maternal age continues to rise in high-income countries.
- Research on adverse birth outcomes at advanced maternal age is mainly focused on fetal death and infant mortality and morbidity.
- It is not known whether older women are also at higher risk of severe maternal morbidity.

**What did the researchers do and find?:**

- We examined maternal mortality and severe morbidity by maternal age.
- The risk of potentially life-threatening morbidity, such as renal failure, shock, amniotic fluid embolism, and cardiac morbidity, increases rapidly among mothers over 39 years old.
- The rate of severe maternal morbidity among women over 50 years old may be higher than the rate of mortality/serious morbidity of their offspring.

**What do these findings mean?:**

- These results should improve counselling to women who contemplate delaying childbirth until their forties and provide useful information to their health care providers.

## Introduction

One of the United Nations’ Millennium Development Goals of 2000 was to reduce maternal mortality by 75% in 15 y [1], a challenge that spurred an interest in maternal mortality and morbidity [2,3]. This challenge was not met by many industrialized countries [4–6]. Moreover, recent reports suggest an increase in maternal deaths in the United States, with the maternal mortality ratio rising from 12 per 100,000 live births in 1990 to 14 per 100,000 live births in 2015 [6–9]. Several explanations have been offered for the increase, including improved ascertainment of maternal deaths, especially those resulting from indirect obstetric causes and late deaths occurring after 42 d postpartum [10], and a temporal increase in chronic health conditions in the child-bearing population. Past decades have seen a rise in the number of parturient women with hypertension, diabetes, chronic heart disease, obesity [11–15], and advanced maternal age [16–21], reflecting an increased complexity of obstetric care, requiring careful prenatal monitoring and timely obstetric interventions.

One of the most remarkable recent demographic changes in industrialized countries has been an upward shift in maternal age [16–21]. In the US, for example, the proportion of births to teenage mothers declined from 12.8% in 1990 to 7.0% in 2013, while the proportion of births to women over 40 y increased from 1.2% to 2.8% [16,17]. The age-specific fertility rate declined from 59.9 births per 1,000 women in 1990 to 24.2 in 2014 for women aged 15–19 y, while the rate doubled among women aged 40–44 y (from 5.5 to 10.6 per 1,000 women) and quadrupled among women aged ≥45 y (from 0.2 to 0.8 births per 1,000 women) [17]. In the United Kingdom, the fertility rate declined from 33.3 per 1,000 women in 1990 to 14.5 in 2015 for women under 20 y but increased from 5.3 to 15.2 per 1,000 women for women ≥40 y over the same period [21]. In 2015, for the first time since 1947, the fertility rate among women aged 40 y or more rose above the rate in women under 20 y in the UK [21].

The effect of older maternal age on elevated rates of cesarean delivery, fetal death, and neonatal mortality and morbidity is well recognized [19,22–28]. While these infant outcomes have been extensively studied using broadly accessible information from birth certificates (e.g., stillbirth and linked live-birth infant death files from the US National Center for Health Statistics) and birth registries, little information is available on the consequences for the health of the mother. This gap leads to clinical uncertainty in risk identification. Our objective was to examine the association between maternal age and severe maternal morbidity using detailed information from a unique link of two population databases—live birth/fetal death certificates and maternal hospitalization files—in Washington State, US. We hypothesized that, besides the well-recognized elevated rates of fetal and neonatal mortality and morbidity at the extremes of maternal age, the risks of severe maternal morbidity would also be significantly increased.

## Methods

All analyses were performed on publicly accessible de-identified data. An exemption from ethics approval was granted by the Department of Social and Health Services of Washington State. This study is reported as per the Strengthening the Reporting of Observational Studies in Epidemiology (STROBE) guidelines (S1 Checklist).

We studied all singleton live births and stillbirths to mothers aged 15–60 y in Washington State from 1 January 2003 to 31 December 2013. We used information from two linked population databases: (a) live birth/fetal death certificates with information on demographics and pregnancy and birth characteristics and (b) hospitalization files including information on specific maternal and infant morbidity. The Birth Events Record Database (BERD) (the database has since been renamed: Linked Birth—CHARS, 1987–2013; http://www.doh.wa.gov/DataandStatisticalReports/VitalStatisticsData/OrderDataFiles#Linked) includes information on maternal characteristics (maternal age, race, education, marital status, body mass index [BMI], chronic hypertension, diabetes mellitus, obstetric history, etc.); pregnancy, labor, and delivery characteristics (gestational diabetes, hypertension in pregnancy, gestational age at delivery, mode of delivery, prolonged labor, etc.); and birth outcomes (fetal and neonatal death, Apgar score at 5 min, birth weight, occurrence of neonatal seizures, neonatal intensive care unit [NICU] admission, etc.). The BERD data were linked with the Comprehensive Hospital Abstract Reporting System (CHARS) database, which includes all hospitalizations in Washington State and up to nine diagnostic and six procedure codes related to each hospitalization episode. We used maternal delivery hospitalization records for information on maternal death and maternal morbidity during delivery and the type of health insurance coverage, and infant birth hospitalization records for detailed information on severe neonatal morbidity and congenital anomalies diagnosed at birth. We did not include multiple pregnancies because the BERD dataset did not provide linkage between births from the same pregnancy (even though they were flagged as twins, triplets, etc.), and their inclusion in our study would therefore compromise the analysis (i.e., mothers of twins would be included twice in the dataset). In addition, multiple pregnancies are clinically distinct from singleton pregnancies (e.g., they have lower average gestational age at delivery, and some complications occur only among multiples, including twin-to-twin transfusion syndrome). Mothers aged <15 y were not included in the study, as they have distinct problems with respect to child abuse, low socioeconomic status, etc.

The originally proposed analysis was to examine maternal age in the following categories: 15–19, 20–24, 25–29 (reference category), 30–34, 35–39, 40–44, and ≥45 y, with additional analyses of the outcomes with higher incidence among women ≥50 y. This categorization is commonly used in the literature, and the age categories span equal numbers of years; maternal age ≥35 y is generally considered advanced maternal age, while maternal age ≥40 y is considered “very advanced” maternal age. In the revised analysis, we included the maternal age category 45–49 y to examine severe morbidities with higher incidence rates, and a category ≥50 y for a composite maternal death/severe morbidity outcome (see below). Maternal death was identified from the CHARS database using the hospital discharge code for death. ICD-9-CM diagnostic and procedure codes were used to identify severe maternal morbidity. Severe maternal morbidity was identified using criteria developed by the Canadian Perinatal Surveillance System [29] based on high case fatality (e.g., sepsis), vital organ function damage (e.g., acute renal failure), high resource utilization (e.g., major surgical procedures), and important adverse sequelae (e.g., peripartum hysterectomy) (see S1 Table). We included additional conditions recognized as severe maternal morbidity by the US Centers for Disease Control and Prevention [30,31]: life-saving procedures such as mechanical ventilation, conversion of cardiac rhythm, etc. Severe maternal morbidity was divided into the following categories: (1) antepartum hemorrhage that required transfusion, e.g., placenta previa, placental abruption; (2) respiratory morbidity, e.g., obstetric pulmonary embolism, respiratory arrest; (3) thromboembolism, e.g., deep venous thrombosis; (4) cerebrovascular morbidity, e.g., cerebral venous thromboembolism, intracranial hemorrhage; (5) all acute cardiac morbidity, e.g., acute myocardial infarction, cardiac arrest, heart failure, peripartum cardiomyopathy; (6) severe postpartum hemorrhage (PPH) requiring transfusion; (7) maternal sepsis, e.g., major puerperal infection, septicemia during labor; (8) renal failure; (9) obstetric shock; (10) complications of anesthesia and obstetric interventions, including shock due to anesthesia, cardiac and pulmonary complications; and (11) severe morbidity denoted by the need for potentially life-saving procedures, e.g., transfusion, hysterectomy, mechanical ventilation. These categories aimed to describe specific groups of severe morbid conditions and were not mutually exclusive. A composite outcome of maternal death/severe morbidity included any of these conditions or procedures; other potentially life-threatening conditions, such as disseminated intravascular coagulation (DIC), eclampsia, uterine rupture, or acute liver failure (see S1 Table); admission to an intensive care unit (ICU); and maternal mortality. In addition, we aimed to describe the maternal age-specific incidence of specific morbidities, such as amniotic fluid embolism (AFE) and peripartum cardiomyopathy, and the rate of admission to an ICU.

Neonatal death was defined as infant death within 28 d after birth. Perinatal mortality was defined as stillbirth (intrapartum or antepartum fetal death after 20 wk of gestation) or neonatal death. Severe neonatal morbidity was identified from the CHARS database using the ICD-9-CM diagnostic codes and included conditions such as bronchopulmonary dysplasia, respiratory distress syndrome, retinopathy of prematurity, intraventricular hemorrhage grade 3 or more, intracranial hemorrhage, neonatal sepsis, necrotizing enterocolitis, and severe birth trauma (S1 Table). The occurrence of any of these conditions, perinatal mortality, or neonatal seizures composed a composite outcome of perinatal death/severe neonatal morbidity. In addition, we compared maternal age-specific incidence of other adverse birth outcomes, including preterm birth (<37 wk) and very preterm birth (<34 wk), small for gestational age (<10th percentile), large for gestational age (>90th percentile), Apgar score < 3 at 5 min, and NICU admission. SGA and LGA percentiles were derived from the US population [32].

Crude odds ratios (ORs) and 95% confidence intervals (CIs) for each maternal age category (relative to 25–29 y) were used to describe the association between maternal age and severe morbidities; we did not perform overall statistical significance tests yielding *p*-values. Logistic regression was used to adjust for demographic and pre-pregnancy characteristics, including race (non-Hispanic white, African-American, Hispanic, Native American, and other—including Asian), marital status (single/widowed/separated versus married/common law), pre-pregnancy BMI, smoking during pregnancy (no versus yes), parity, maternal education (high school graduate or more education versus less than high school graduation), type of health insurance (Medicaid, private, self-pay, other), fetal sex (female versus male), and year of childbirth. Parity and BMI were modelled using splines with a maximum of three knots. Sensitivity analyses were performed to adjust for underlying medical conditions (chronic hypertension, diabetes mellitus) and assisted conception, to examine their impact on the results. In addition, other pregnancy and labor characteristics (e.g., mode of delivery, labor induction) were added as covariates. These factors can be considered as mediators on the causal pathway between maternal age and maternal severe morbidity and adverse perinatal outcomes, and require strong assumptions about the directionality of associations, and were therefore not adjusted for in the main analyses. However, adjusting for these factors adds additional insight into the risks of severe maternal morbidity among older women who do not have any apparent chronic condition or indication for cesarean delivery.

Besides ORs and adjusted ORs (AORs) and their 95% CIs, we also present crude and adjusted risk differences (ARDs) with their 95% CIs to convey the magnitude of absolute risk increases. The adjusted age-specific risk difference indicates the additional number of mothers with adverse outcome above the baseline rate that would be expected if the same demographic and pre-pregnancy characteristics as the reference group (25–29 y) were present.

Analyses were carried out using SAS 9.4. Missing values for BMI (approximately 10%) were imputed using the multiple imputation procedure (PROC MI) in SAS. Other missing values that constituted <3% of the total for a factor are not included in the tables, but are presented in S2 Table. Except for BMI missing values (which were imputed), complete-case multivariable analyses were performed.

## Results

Overall, 952,212 mothers gave birth (live or stillbirth) in Washington State between 1 January 2003 and 31 December 2013. We excluded births that occurred out of state, multiple births, births before 20 wk gestation, births to females aged <15 or >60 y (35,598 mothers, 3.7%), births that occurred out of hospital (24,716 mothers, 2.6%), and births that could not be matched with hospital records (64,609 mothers, 6.8%). The remaining 828,269 births were included in the study. Women who delivered out of hospital or whose birth could not be matched with hospital records were not substantially different from those included in the study, except for type of health insurance (they were more likely to have “other” insurance—other government insurance, student insurance, Indian Health Care, other health insurance programs, or no insurance; S2 Table).

The largest proportion of births was to mothers 25–29 y of age (28.9%), 25.3% of births were to mothers aged 30–35 y, and 22.5% of births were to mothers aged 20–24 y. Births to teenage mothers comprised 7.6%, while births to older mothers aged 35–39, 40–44, and ≥45 y comprised 12.7%, 2.8%, and 0.2% of all singleton births, respectively. Teenage mothers were more likely to be Hispanic or African-American, while older mothers (35–39, 40–44, and ≥45 y) were more likely to be non-Hispanic white or “other” than were younger mothers (Table 1).

**Table 1:** Maternal age-specific demographic and pregnancy characteristics, singleton births, Washington State, US, 2003–2013.

Characteristic	Maternal age						
	15–19 y (*n = * 62,904)	20–24 y (*n = * 186,537)	25–29 y (*n = * 239,319)	30–34 y (*n = * 209,936)	35–39 y (*n = * 104,985)	40–44 y (*n = * 23,180)	≥45 y (*n = * 1,408)
**Race**							
Non-Hispanic white	40,533 (64.7)	132,907 (71.5)	174,700 (73.2)	152,104 (72.7)	75,236 (71.9)	16,565 (71.8)	1,012 (72.2)
African-American	3,253 (5.2)	8,797 (4.7)	9,206 (3.9)	6,984 (3.3)	3,691 (3.5)	968 (4.2)	93 (6.6)
Native American	2,829 (4.5)	5,568 (3.0)	4,418 (1.9)	2,588 (1.2)	1,139 (1.1)	241 (1.0)	6 (0.4)
Hispanic	13,579 (21.7)	27,953 (15.0)	26,397 (11.1)	18,492 (8.8)	8,800 (8.4)	1,937 (8.4)	102 (7.3)
Other	2,461 (3.9)	10,728 (5.8)	23,877 (10.0)	29,110 (13.9)	15,749 (15.1)	3,353 (14.5)	189 (13.5)
**Maternal education less than high school**	2,482 (4.0)	6,983 (3.7)	9,274 (3.9)	8,494 (4.1)	4,794 (4.6)	1,239 (5.4)	90 (6.4)
**Smoking during pregnancy**	10,365 (16.6)	29,038 (15.7)	22,478 (9.5)	11,599 (5.6)	4,866 (4.7)	1,166 (5.1)	46 (3.3)
**Not married**	52,238 (83.9)	29,038 (54.5)	22,478 (27.6)	11,599 (16.4)	4,866 (15.0)	1,166 (17.7)	246 (17.7)
**Medical insurance**							
Medicaid	45,416 (72.2)	109,433 (58.7)	89,201 (37.3)	52,294 (24.9)	23,144 (22.5)	5,579 (24.1)	329 (23.4)
Self-pay	514 (0.2)	1,548 (0.8)	2,156 (0.9)	2,143 (1.0)	1,149 (1.1)	275 (1.2)	28 (2.0)
Private	10,379 (16.5)	54,428 (29.2)	127,256 (53.2)	140,398 (66.9)	73,327 (69.9)	15,640 (67.5)	930 (66.1)
Other (including government)^a^	4,987 (7.9)	15,923 (8.5)	13,451 (5.6)	8,248 (3.9)	3,576 (3.4)	806 (3.5)	54 (3.8)
**Parity**							
Nullipara	51,560 (82.4)	95,289 (51.4)	90,681 (38.1)	65,638 (31.4)	26,904 (25.8)	5,323 (23.2)	384 (27.7)
Multipara (1–3 previous births)	9,802 (15.6)	85,499 (45.8)	135,387 (56.6)	126,691 (60.4)	65,033 (62.0)	13,318 (57.5)	636 (45.2)
Grand multipara (≥4 previous births)	1,186 (1.9)	4,642 (2.5)	12,051 (5.0)	16,560 (7.9)	12,380 (11.8)	4,334 (18.7)	369 (26.2)
**Body mass index (kg/m**^**2**^**)**							
Underweight (<18.5)	3,247 (5.2)	6,979 (3.7)	6,425 (2.7)	4,843 (2.3)	2,096 (2.0)	408 (1.8)	21 (1.5)
Normal (18.5–24.9)	30,641 (48.7)	76,922 (41.2)	99,556 (41.6)	91,332 (43.5)	44,586 (42.5)	9,292 (40.1)	539 (38.3)
Overweight (25.0–29.9)	13,251 (21.1)	42,713 (22.9)	56,258 (23.5)	48,809 (23.3)	24,871 (23.7)	5,714 (24.7)	375 (26.6)
Obese (≥30)	9,007 (14.3)	41,132 (22.1)	53,752 (22.5)	43,535 (20.7)	21,885 (20.9)	5,154 (22.2)	287 (20.4)
Missing	6,758 (10.7)	18,791 (10.1)	23,328 (9.8)	21,417 (10.2)	11,547 (11.0)	2,612 (11.3)	186 (13.2)
**Assisted conception**	5 (<0.1)	223 (0.1)	1,330 (0.6)	2,462 (1.2)	2,182 (2.1)	948 (4.2)	252 (18.3)
**Diabetes mellitus (type 1 and 2)**	187 (0.3)	994 (0.5)	1,822 (0.8)	2,205 (1.1)	1,592 (1.5)	483 (2.1)	24 (1.7)
**Chronic hypertension**	255 (0.4)	1,360 (0.7)	2,710 (1.2)	3,097 (1.5)	2,277 (2.2)	799 (3.5)	71 (5.2)
**Neonatal sex (male)**	32,198 (51.2)	95,679 (51.3)	123,025 (51.4)	107,840 (51.4)	53,808 (51.3)	11,747 (50.7)	710 (50.4)
**Congenital anomalies (any)**	3,769 (6.0)	10,634 (5.7)	13,775 (5.8)	12,813 (6.1)	6,934 (6.6)	1,673 (7.2)	106 (8.0)

Data given as *n* (percent). The percentages for some characteristics do not add up to 100% due to missing values; missing values <3% are not shown.

^a^Other medical insurance includes other government insurance, student insurance, Indian Health Care, other programs, or no insurance.

The proportions of non-married, nulliparous, and underweight women declined with maternal age, while the proportions of women with assisted conception, obesity, diabetes mellitus, and chronic hypertension increased with age (Table 1). Cesarean delivery was more common among older women—especially primary and repeat cesarean delivery without labor (Table 2).

**Table 2:** Maternal age-specific labor and delivery characteristics, singleton births, Washington State, US, 2003–2013.

Characteristic	Maternal age						
	15–19 y (*n = * 62,904)	20–24 y (*n = * 186,537)	25–29 y (*n = * 239,319)	30–34 y (*n = * 209,936)	35–39 y (*n = * 104,985)	40–44 y (*n = * 23,180)	≥45 y (*n = * 1,408)
**Previous preterm or SGA baby**	408 (0.7)	3,861 (2.1)	6,230 (2.6)	5,925 (2.9)	3,347 (3.3)	757 (3.3)	48 (3.5)
**Fetal presentation**							
Cephalic	59,615 (94.8)	176,484 (94.6)	225,722 (94.3)	195,873 (93.3)	96,958 (92.4)	21,155 (91.3)	1,239 (88.0)
Breech	1,417 (2.3)	4,485 (2.4)	6,602 (2.8)	6,947 (3.3)	3,941 (3.8)	1,060 (4.6)	109 (7.7)
Other	451 (0.7)	1,356 (0.7)	1,618 (0.7)	1,567 (0.8)	901 (0.9)	252 (1.1)	14 (1.0)
Unknown	1,421 (2.3)	4,212 (2.3)	5,377 (2.2)	5,549 (2.6)	3,185 (3.0)	713 (3.1)	46 (3.3)
**Cesarean delivery**							
Primary with trial of labor	6,329 (10.1)	16,978 (9.1)	20,936 (8.7)	18,287 (8.7)	9,572 (9.1)	2,331 (10.1)	162 (11.5)
Primary without trial of labor	3,947 (6.3)	13,676 (7.3)	20,443 (8.5)	21,712 (10.4)	13,478 (12.9)	3,660 (15.8)	322 (22.9)
Repeat with trial of labor	97 (0.2)	900 (0.5)	1,626 (0.7)	1,811 (0.9)	1,098 (1.1)	268 (1.2)	14 (1.0)
Repeat without trial of labor	1,018 (1.6)	11,161 (6.0)	21,040 (8.8)	23,666 (11.3)	14,666 (14.0)	3,526 (15.2)	174 (12.4)
**Vaginal delivery**							
Spontaneous	46,671 (74.2)	131,749 (70.7)	158,519 (66.3)	129,023 (61.5)	58,566 (55.8)	11,725 (50.6)	649 (46.1)
VBAC	164 (0.3)	1,727 (0.9)	3,562 (1.5)	3,858 (1.8)	2,237 (2.1)	552 (2.4)	17 (1.2)
Forceps	553 (0.9)	1,243 (0.7)	1,701 (0.7)	1,469 (0.7)	650 (0.6)	124 (0.5)	11 (0.8)
Forceps VBAC	6 (<0.1)	39 (<0.1)	59 (<0.1)	60 (<0.1)	40 (<0.1)	7 (<0.1)	—
Vacuum	4,062 (6.5)	8,824 (4.7)	10,960 (4.6)	9,500 (4.5)	4,360 (4.2)	907 (3.9)	57 (4.1)
Vacuum VBAC	21 (<0.1)	169 (0.1)	353 (0.1)	431 (0.2)	253 (0.2)	62 (0.3)	2 (0.1)
**Previous cesarean delivery**	1,308 (2.1)	13,999 (7.5)	26,643 (11.1)	29,838 (14.2)	18,301 (17.5)	4,417 (19.1)	207 (14.7)
1 prior delivery	1,217 (1.9)	11,290 (6.1)	19,739 (8.3)	21,921 (10.5)	13,145 (12.6)	3,199 (13.8)	158 (11.2)
2 or more prior deliveries	83 (0.1)	2,652 (1.4)	6,779 (2.8)	7,723 (3.7)	5,029 (4.8)	1,180 (5.1)	48 (3.4)
**Labor induction**	14,296 (23.1)	44,071 (24.0)	55,585 (23.6)	44,688 (21.7)	21,207 (20.7)	4,777 (21.2)	305 (22.6)
**PROM (>12 h)**	3,213 (5.2)	8,976 (4.9)	11,800 (5.0)	10,848 (5.3)	5,462 (5.4)	1,241 (5.6)	85 (6.3)
**Precipitous labor (<3 h)**	1,072 (1.7)	4,623 (2.5)	7,198 (3.1)	7,116 (3.5)	3,777 (3.7)	807 (3.6)	47 (3.5)
**Prolonged labor**	1,734 (2.8)	4,294 (2.4)	5,165 (2.2)	4,071 (2.0)	1,988 (2.0)	424 (1.9)	27 (2.0)
**Hypertension in pregnancy**	3,794 (6.0)	10,332 (5.5)	12,416 (5.2)	10,148 (4.8)	5,297 (5.1)	1,374 (5.9)	113 (8.0)
**Gestational diabetes**	1,069 (1.7)	6,248 (3.4)	13,777 (5.8)	17,102 (8.2)	11,505 (11.0)	3,268 (14.1)	221 (15.7)
**Chorioamnionitis**	1,615 (2.6)	3,986 (2.2)	4,613 (2.0)	3,628 (1.8)	1,576 (1.5)	351 (1.6)	20 (1.5)

Data given as *n* (percent). Information on cesarean delivery with and without labor was abstracted from charts (and included in the Birth Events Record Database).

PROM, premature rupture of membranes; SGA, small for gestational age; VBAC, vaginal birth after cesarean delivery.

The incidence of gestational diabetes increased markedly with maternal age, from 1.7% among teen mothers to 15.7% among the oldest mothers (45–60 y). Nearly all women had more than two prenatal care visits, with little variation between teenage mothers (97.7%) and the oldest mothers (98.4%).

**Fig 1:**
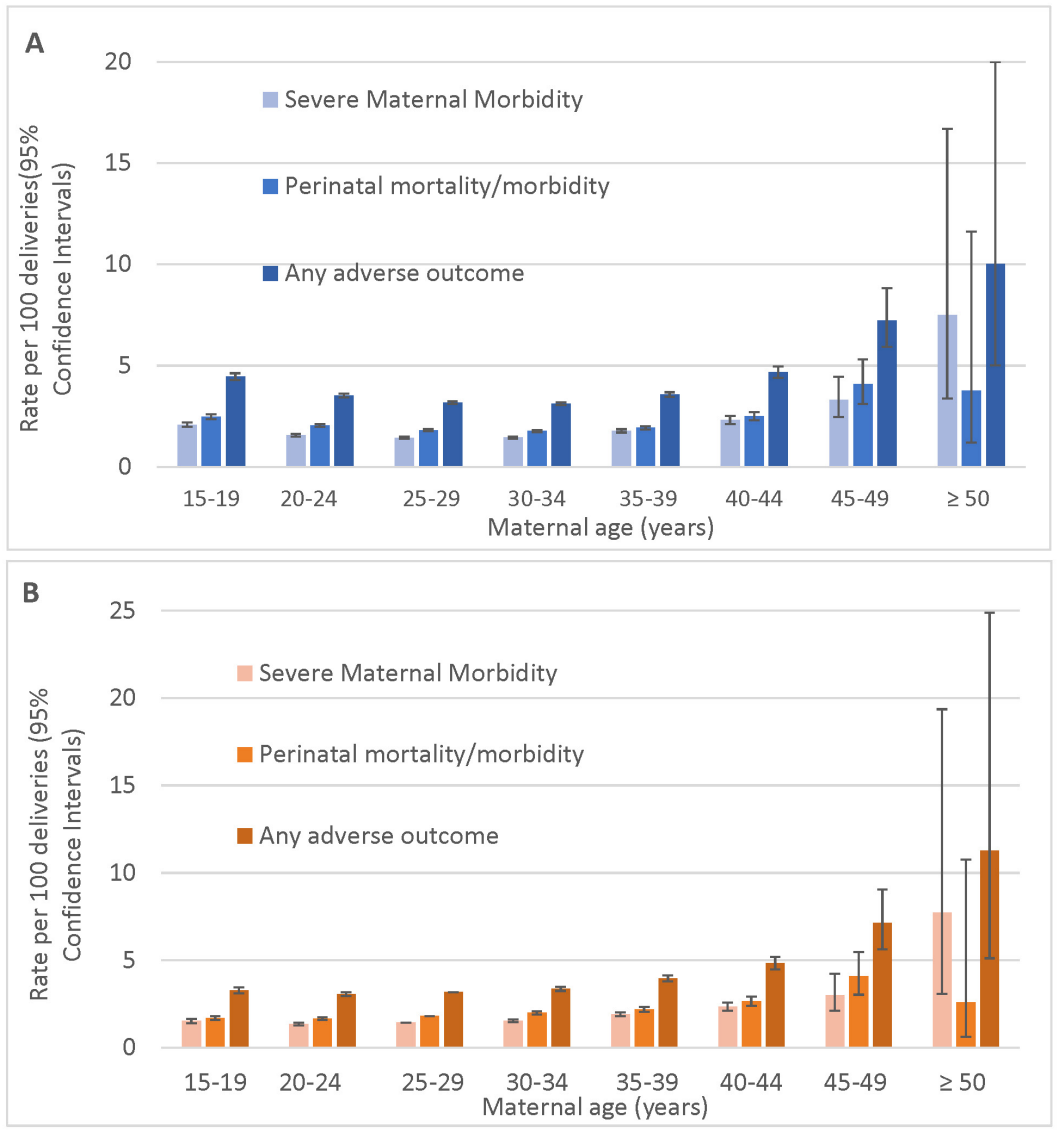
Age-specific rates of severe maternal morbidity and perinatal mortality/severe neonatal morbidity. (A) Unadjusted rates and (B) rates adjusted for demographic and pre-pregnancy factors; singleton births, Washington State, US, 2003–2013. Bars show 95% CIs.

The overall rate of severe maternal morbidity/mortality was 1.6 per 100 deliveries. Most severe maternal morbidity showed a J-shaped association with maternal age (Fig 1A; Table 3), with elevated rates among teenage mothers, low rates among mothers 20–34 y of age, and a sharp increase among mothers over 39 y of age.

**Table 3:** Maternal mortality and severe maternal morbidity and rate per 10,000 singleton births, Washington State, US, 2003–2013.

Outcome	Maternal age						
	15–19 y (*n = * 62,904)	20–24 y (*n = * 186,537)	25–29 y (*n = * 239,319)	30–34 y (*n = * 209,936)	35–39 y (*n = * 104,985)	40–44 y (*n = * 23,180)	≥45 y (*n = * 1,408)
**Death**	4 (0.6)	12 (0.6)	12 (0.5)	16 (0.8)	7 (0.7)	2 (0.9)	—
**Antepartum hemorrhage/abruption**^a^	35 (5.6)	122 (6.5)	206 (8.6)	161 (7.7)	123 (11.7)	41 (17.7)	4 (28.4)
**Respiratory morbidity**	73 (11.6)	204 (10.9)	252 (10.5)	228 (10.9)	153 (14.6)	54 (23.3)	2 (14.2)
Obstetric embolism	8 (1.3)	30 (1.6)	45 (1.9)	44 (2.1)	21 (2.0)	11 (4.8)	—
AFE	2 (0.3)	6 (0.3)	8 (0.3)	12 (0.6)	9 (0.9)	7 (3.0)	—
Blood clot embolism (VTE)	6 (1.0)	21 (1.1)	34 (1.4)	29 (1.4)	9 (0.9)	3 (1.3)	—
**Thromboembolism/DVT**	32 (5.1)	82 (4.4)	136 (5.7)	114 (5.4)	67 (6.4)	21 (8.5)	—
**Cerebrovascular morbidity**	12 (1.9)	43 (2.3)	58 (2.4)	56 (2.7)	30 (2.9)	13 (5.6)	1 (7.1)
Cerebral venous thrombosis	5 (0.8)	8 (0.4)	31 (1.3)	22 (1.1)	9 (0.9)	4 (1.7)	—
Cerebrovascular disorders in puerperium	3 (0.5)	13 (0.7)	23 (2.0)	15 (0.7)	13 (1.2)	6 (2.6)	1 (7.1)
**Acute cardiac morbidity**	17 (2.7)	77 (4.1)	112 (4.7)	116 (5.5)	83 (7.9)	36 (15.5)	1 (7.1)
Cardiac arrest/failure/MI	12 (1.9)	37 (2.0)	47 (2.0)	49 (2.3)	35 (3.3)	19 (8.2)	—
Peripartum cardiomyopathy	4 (0.6)	16 (0.9)	20 (0.8)	30 (1.4)	10 (1.0)	7 (3.0)	1 (7.1)
**Severe PPH**^a^	344 (54.7)	797 (42.7)	913 (38.2)	831 (39.6)	486 (46.3)	116 (50.0)	16 (113.6)
PPH with coagulation defects	65 (10.3)	175 (9.4)	231 (9.7)	210 (10.0)	129 (12.3)	39 (16.8)	2 (14.2)
**Maternal sepsis**	383 (60.9)	741 (39.7)	672 (28.1)	509 (24.3)	272 (25.9)	68 (29.3)	2 (14.2)
Puerperal sepsis	372 (59.1)	696 (37.3)	632 (26.4)	476 (22.7)	251 (23.9)	63 (27.2)	1 (7.1)
**Renal failure**	13 (2.1)	25 (1.3)	41 (1.7)	37 (1.8)	29 (2.8)	10 (4.3)	4 (28.4)
**Obstetric shock**	8 (1.3)	16 (0.9)	28 (1.2)	42 (2.0)	35 (3.3)	10 (4.3)	1 (7.1)
**Complications**^b^	79 (12.6)	228 (12.2)	387 (16.2)	401 (19.1)	260 (24.8)	62 (26.8)	9 (63.9)
**Potentially life-saving procedures**	641 (101.9)	1,434 (76.9)	1,733 (72.4)	1,543 (73.5)	962 (91.6)	297 (128.1)	31 (220.2)
Hysterectomy	9 (1.4)	35 (1.9)	129 (5.4)	170 (8.1)	154 (14.7)	53 (22.9)	11 (78.1)
Blood/blood products transfusion	598 (95.1)	1,270 (68.1)	1,427 (59.6)	1,197 (57.0)	702 (66.9)	207 (89.3)	21 (149.2)
Respiratory (assisted ventilation)	20 (3.2)	80 (4.3)	89 (3.7)	84 (4.0)	73 (7.0)	23 (9.9)	3 (21.3)
**DIC**	10 (1.6)	27 (1.5)	52 (2.2)	64 (3.1)	38 (3.6)	14 (6.0)	4 (28.4)
**Uterine rupture**	14 (2.2)	75 (4.0)	137 (5.7)	140 (6.7)	110 (10.5)	29 (12.5)	1 (7.1)
**ICU admission**	62 (10.0)	130 (7.1)	195 (8.3)	176 (8.6)	122 (11.9)	37 (16.4)	11 (80.2)
**Maternal death/severe morbidity**	1,307 (207.8)	2,913 (156.2)	3,431 (143.4)	3,022 (144.0)	1,863 (177.5)	535 (230.8)	50 (355.1)

Data given as *n* (rate per 10,000 singleton births). Categories overlap; the composite outcome maternal death/severe morbidity includes all of the conditions listed in this table and in S1 Table: eclampsia, acute liver failure, etc.

^a^With transfusion.

^b^Complications of anesthesia and obstetric interventions.

AFE, amniotic fluid embolism; DIC, disseminated intravascular coagulation; DVT, deep venous thrombosis; ICU, intensive care unit; MI, myocardial infarction; PPH, postpartum hemorrhage; VTE, venous thromboembolism.

An exception was the rate of maternal sepsis, which was highest among teen mothers (65.8 per 10,000 deliveries) and declined with maternal age (to 14–35 per 10,000 deliveries among mothers over 34 y of age). Some severe morbid conditions had the lowest rate among teenage mothers, with the rate increasing with maternal age, e.g., obstetric embolism, AFE, acute cardiac morbidity, uterine rupture, and hysterectomy. The rates of severe PPH, renal failure, DIC, complications of obstetric interventions, and potentially life-saving procedures increased rapidly in women above 39 y. Similarly, the rate of ICU admission increased sharply among mothers over 44 y of age (80.2 per 10,000 deliveries, compared to 7.1 per 10,000 deliveries among mothers 20–24 y). The rate of the composite outcome of maternal death or severe morbidity was 2.1% among teen mothers, 1.5% among mothers aged 25–29 y, and 3.6% among mothers over 44 y of age. The J-shaped association was also evident between maternal age and fetal/infant outcomes (S3 Table), except for the rates of large for gestational age and macrosomia, which were lowest among teen mothers.

Teen mothers were not at significantly elevated risk of severe maternal morbidity compared to mothers aged 25–29 y after adjustment for maternal demographic and pre-pregnancy factors (Table 4; S1 Fig). They were significantly less likely to experience cerebrovascular morbidity, acute cardiac morbidity, and complications of obstetric interventions. However, teen mothers were at elevated risk of sepsis (AOR = 1.2, 95% CI 1.1–1.4). After adjustment, the ORs for most types of severe maternal morbidity remained significantly increased among women over 39 y of age. In particular, mothers 40–44 y had a 3-fold higher risk of shock (AOR = 2.9, 95% CI 1.3–6.6), an 8-fold increased risk of AFE (AOR = 8.0, 95% CI 2.7–23.7), and considerably elevated acute cardiac morbidity (AOR = 3.8, 95% CI 2.5–5.7), including cardiomyopathy (AOR = 4.7, 95% CI 1.9–11.4; Table 4). Mothers 45–49 y had higher risk of renal failure (AOR = 15.9, 95% CI 4.8–52), complications of obstetric interventions (AOR = 4.7, 95% CI 2.3–9.5), and ICU admission (AOR = 4.8, 95% CI 2.0–11.9).

**Table 4:** The association between maternal age and severe maternal morbidity and adverse birth outcomes among singleton births, Washington State, US, 2003–2013.

Outcome	Measure	OR^a^ by maternal age						AOR^b^ by maternal age					
		15–19 y	20–24 y	30–34 y	35–39 y	40–44 y	45–49 y	15–19 y	20–24 y	30–34 y	35–39 y	40–44 y	45–49 y
**Severe maternal morbidity**													
Antepartum hemorrhage^c^	OR or AOR	0.7	0.8	0.9	1.4	2.1	3.5	0.8	0.7	0.8	1.2	1.5	1.6
	95% CI	0.4–0.9	0.6–0.9	0.7–1.1	1.1–1.7	1.5–2.9	1.3–9.4	0.5–1.1	0.6–0.9	0.7–1.1	1.0–1.6	1.0–2.2	0.4–6.6
Respiratory morbidity	OR or AOR	1.1	1.0	1.0	1.4	2.2		0.7	0.8	1.1	1.4	2.5	
	95% CI	0.8–1.4	0.9–1.2	0.9–1.2	1.1–1.7	1.73.0		0.5–1.0	0.7–1.0	0.9–1.4	1.1–1.8	1.8–3.4	
AFE	OR or AOR	1.0	1.0	1.7	2.6	9.0		0.8	0.7	1.6	2.3	8.0	
	95% CI	0.2–4.5	0.3–2.8	0.7–4.2	1.0–6.6	3.3–25		0.1–4.0	0.2–2.4	0.6–4.0	0.8–6.2	2.7–23.7	
Thromboembolism/DVT	OR or AOR	0.9	0.8	1.0	1.1	1.6		0.8	0.7	1.0	1.2	1.6	
	95% CI	0.6–1.3	0.6–1.0	0.7–1.2	0.8–1.5	1.0–2.5		0.5–1.2	0.6–1.0	0.8–1.3	0.9–1.6	1.0–2.6	
Cerebrovascular morbidity	OR or AOR	0.8	1.0	1.1	1.2	2.3	3.1	0.3	0.7	1.4	1.6	3.2	4.8
	95% CI	0.4–1.5	0.6–1.4	0.8–1.6	0.8–1.8	1.3–4.2	0.4–22	0.2–0.7	0.4–1.0	0.9–2.0	1.0–2.5	1.7–6.0	0.7–34.6
All acute cardiac morbidity	OR or AOR	0.6	0.9	1.2	1.7	3.3		0.5	0.8	1.3	1.8	3.8	
	95% CI	0.3–1.0	0.7–1.2	0.9–1.5	1.3–2.2	2.3–4.8		0.3–0.9	0.6–1.0	1.0–1.7	1.3–2.5	2.5–5.7	
Cardiomyopathy	OR or AOR	0.8	1.0	1.7	1.1	3.6		0.6	0.7	2.0	1.0	4.7	
	95% CI	0.3–2.2	0.5–2.0	1.0–3.0	0.5–2.4	1.5–8.6		0.2–2.0	0.3–1.5	1.1–3.6	0.4–3.6	1.9–11.4	
Severe PPH^c^	OR or AOR	1.5	1.1	1.0	1.2	1.3	2.5	1.2	1.0	1.0	1.2	1.2	1.9
	95% CI	1.3–1.7	1.0–1.2	0.9–1.1	1.1–1.3	1.1–1.6	1.4–4.5	1.0–1.4	0.9–1.1	0.9–1.1	1.1–1.4	0.9–1.5	0.9–3.8
Maternal sepsis	OR or AOR	2.2	1.4	0.9	0.9	1.0		1.2	1.1	1.0	1.1	1.2	
	95% CI	1.9–2.5	1.3–1.6	0.8–1.0	0.8–1.1	0.8–1.3		1.1–1.4	1.0–1.3	0.8–1.1	0.9–1.2	0.9–1.6	
Renal failure	OR or AOR	1.2	0.8	1.0	1.6	2.5	13.2	1.1	0.7	1.1	1.7	2.7	15.9
	95% CI	0.6–2.3	0.5–1.3	0.7–1.6	1.0–2.6	1.3–5.0	4.1–42.7	0.5–2.2	0.4–1.3	0.7–1.7	1.0–2.8	1.3–5.6	4.8–52
Obstetric shock	OR or AOR	1.1	0.7	1.7	2.9	3.7		0.8	0.8	1.6	2.6	2.9	
	95% CI	0.5–2.4	0.4–1.4	1.1–2.8	1.7–4.7	1.8–7.6		0.3–2.1	0.4–1.4	1.0–2.7	1.5–4.5	1.3–6.6	
Complications^d^	OR or AOR	0.8	0.8	1.2	1.5	1.7	4.2	0.6	0.7	1.3	1.7	1.6	4.7
	95% CI	0.6–1.0	0.6–0.9	1.0–1.4	1.3–1.8	1.3–2.2	2.2–8.2	0.5–0.8	0.5–0.8	1.1–1.5	1.4–2.0	1.2–2.2	2.3–9.5
DIC	OR or AOR	0.7	0.7	1.4	1.7	2.8		0.9	0.7	1.3	1.3	1.7	
	95% CI	0.4–1.4	0.4–1.1	1.0–2.0	1.1–2.5	1.5–5.0		0.4–1.9	0.4–1.2	0.91.9	0.8–2.1	0.8–3.5	
Potentially life-saving procedures	OR or AOR	1.4	1.1	1.0	1.3	1.8	3.1	1.1	0.9	1.0	1.3	1.6	2.2
	95% CI	1.3–1.5	1.0–1.1	0.9–1.1	1.2–1.4	1.6–2.0	2.1–4.4	1.0–1.2	0.9–1.0	1.0–1.1	1.2–1.4	1.4–1.9	1.4–3.5
ICU admission	OR or AOR	1.2	0.9	1.0	1.4	2.0	8.4	0.9	0.7	1.0	1.5	1.7	4.8
	95% CI	0.9–1.6	0.7–1.1	0.8–1.3	1.1–1.8	1.4–2.8	4.3–16.5	0.6–1.2	0.6–0.9	0.8–1.3	1.1–1.9	1.2–2.6	2.0–11.9
Maternal death/severe morbidity	OR or AOR	1.5	1.1	1.0	1.2	1.6	2.3	1.1	0.9	1.1	1.3	1.6	2.0
	95% CI	1.4–1.6	1.0–1.1	1.0–1.0	1.2–1.3	1.5–1.8	1.7–3.1	1.0–1.1	0.9–1.0	1.0–1.1	1.3–1.4	1.5–1.8	1.4–2.9
**Fetal/infant mortality and morbidity**													
Fetal death	OR or AOR	1.3	1.1	1.1	1.3	1.8	3.9	1.0	1.0	1.2	1.4	1.7	3.4
	95% CI	1.1–1.4	1.0–1.2	1.0–1.2	1.2–1.4	1.6–2.1	2.5–5.9	0.9–1.2	0.9–1.1	1.1–1.3	1.3–1.6	1.4–2.1	2.0–5.7
Neonatal death	OR or AOR	1.7	1.1	1.0	1.1	1.6	3.0	1.5	1.0	1.1	1.3	1.8	2.6
	95% CI	1.5–2.0	1.0–1.2	0.8–1.1	0.9–1.2	1.3–2.0	1.5–5.8	1.2–1.7	0.9–1.1	1.0–1.3	1.0–1.5	1.4–2.3	1.2–5.9
Perinatal death	OR or AOR	1.4	1.1	1.0	1.2	1.8	3.6	1.2	1.0	1.2	1.4	1.7	3.2
	95% CI	1.3–1.6	1.0–1.2	1.0–1.1	1.1–1.3	1.5–2.0	2.5–5.1	1.0–1.3	0.9–1.1	1.1–1.3	1.3–1.5	1.5–2.0	2.1–4.9
Severe neonatal morbidity^e^	OR or AOR	1.4	1.2	1.0	1.0	1.2	1.6	0.9	1.0	1.0	1.1	1.3	1.8
	95% CI	1.3–1.5	1.0–1.2	0.9–1.0	0.9–1.0	1.1–1.3	1.0–2.4	0.9–1.0	0.9–1.0	1.0–1.1	1.0–1.2	1.1–1.4	1.2–2.8
NICU admission	OR or AOR	1.1	1.1	1.0	1.1	1.3	1.8	0.9	1.0	1.1	1.2	1.4	1.8
	95% CI	1.1–1.2	1.0–1.1	1.0–1.0	1.1–1.1	1.2–1.3	1.5–2.2	0.9–0.9	0.9–1.0	1.0–1.1	1.1–1.2	1.3–1.4	1.4–2.2
Perinatal death/severe neonatal morbidity^e^	OR or AOR	1.4	1.1	1.0	1.1	1.4	2.3	1.0	1.0	1.1	1.2	1.4	2.2
	95% CI	1.3–1.5	1.1–1.2	0.9–1.0	1.0–1.1	1.3–1.5	1.8–3.0	0.9–1.0	0.9–1.0	1.0–1.1	1.1–1.3	1.3–1.6	1.7–3.0
**Perinatal or maternal death/severe morbidity**	OR or AOR	1.4	1.1	1.0	1.1	1.5	2.4	1.0	1.0	1.1	1.2	1.5	2.2
	95% CI	1.4–1.5	1.1–1.2	1.0–1.0	1.1–1.2	1.4–1.6	1.9–2.9	0.9–1.1	0.9–1.0	1.0–1.1	1.2–1.3	1.4–1.6	1.8–2.9

Categories overlap; the composite outcome maternal death/severe morbidity includes all of the conditions listed in Table 1 and S1 Table: eclampsia, acute liver failure, etc.

^a^Relative to women aged 25–29 y.

^b^Adjusted for race, marital status, body mass index, drug use, smoking, parity, maternal education, type of health insurance, year of childbirth, and fetal sex.

^c^With transfusion.

^d^Complications of anesthesia and obstetric interventions.

^e^Includes respiratory distress syndrome, retinopathy of prematurity, intraventricular hemorrhage (grade 3 or more), intracranial hemorrhage, sepsis, necrotizing enterocolitis, severe birth trauma, and seizures.

AFE, amniotic fluid embolism; AOR, adjusted odds ratio; DIC, disseminated intravascular coagulation; DVT, deep venous thrombosis; ICU, intensive care unit; NICU, neonatal intensive care unit; OR, odds ratio; PPH, postpartum hemorrhage.

While the unadjusted absolute rates of adverse maternal outcomes continued to increase with age among women over 39 y of age, the rates of adverse perinatal outcomes plateaued at maternal age ≥45 y (Fig 1A). The adjusted rates of mortality/severe morbidity were substantially lower (Fig 1B). The absolute rates of fetal and neonatal death and morbidity were elevated among teen mothers, and the neonatal death rate remained higher after adjustment for demographic and pre-pregnancy factors, while the rate of NICU admission was significantly lower among teen mothers after adjustment.

Additional analyses showed that severe maternal morbidity and/or mortality increased dramatically among the oldest mothers (≥50 y, *n = * 80). The AOR compared to mothers 25–29 y increased from 1.6 at 40–44 y (95% CI 1.5–1.8) to 2.0 at 45–49 y (95% CI 1.4–2.9), and to 5.2 at 50 y or more (95% CI 2.1–13.1). Such a trend among mothers ≥50 y was also observed for a composite outcome of any perinatal or maternal death/severe morbidity (AOR = 3.6, 95% CI 1.6–7.8).

Sensitivity analyses revealed that the association between maternal age 45–49 y and some severe maternal morbidities attenuated slightly after adjustment for chronic conditions (diabetes mellitus and chronic hypertension) and assisted conception; the AOR for renal failure was 11.4 (95% CI 3.3–39.3). Additional adjustment for labor characteristics and mode of delivery (S4 Table) further attenuated the association of age with some morbidities; however, a significant increase in many severe morbidities remained among women aged 40 y or more. The risk of renal failure remained significantly elevated among those ≥45 y even after additional adjustment for pre-pregnancy and pregnancy hypertension and for preeclampsia (including eclampsia and superimposed preeclampsia and eclampsia).

The adjusted risk difference (ARD) in overall severe maternal morbidity compared to mothers 25–29 y and those 45–49 y was 1.6% (95% CI 0.7%–2.8%) and increased to 6.4% for those ≥50 y (95% CI 1.7%–18.2%; S5 Table). The ARD for the composite outcome of any perinatal or maternal death/severe morbidity was 4.0% for women 45–49 y (95% CI 2.4%–5.9%) and 8.1% for women ≥50 y (95% CI 1.9%–21.6%).

## Discussion

Our results show elevated rates of severe maternal morbidity at the extremes of maternal age. While the elevated risk among teenage mothers was mostly due to an increased rate of sepsis, rates of all other causes of severe maternal morbidity were elevated among older mothers (≥40 y). The absolute rates and ORs were lower after adjustment for demographic and pre-pregnancy factors but remained elevated for sepsis among teenage mothers and for all other morbidities among older women. Our results confirm that perinatal mortality and neonatal morbidity are elevated among teenage mothers and older mothers, as compared with mothers aged 25–29 y [22–25]. The association between teenage motherhood and most adverse perinatal outcomes disappeared after adjustment for maternal demographic factors, with the exception of the neonatal death rate, which remained 50% higher.

Incidence rates of maternal morbidity in industrialized countries vary greatly depending on the definition of “morbidity,” ranging from 0.4% to 31.1% [29–31,33–40], with the highest rates including any morbidity outside a normal delivery [40]. In the US, the population-based incidence of severe maternal morbidity increased from 0.73% of hospital deliveries in 1998/1999 to 1.63% in 2010/2011 [30,34], while hospital-based incidence rates ranged from 0.51% to 2.45% in 2012/2013 [31]. The incidence rate of severe maternal morbidity has been reported as 1.4% in both Canada (in 2007) [29] and Australia (in 2004) [35], 0.7% in the Netherlands (in 2004–2006) [36], 0.5% in England (in 2012/2013) [37], and 0.4% in Scotland (in 2001/2002) [38]. These differences in severe morbidity rates are mainly due to varying definitions of severe morbidity [29–31,33,36–40]. However, discrepancies may also arise due to differences in the study populations, which can be defined geographically (including all births in the country/region) [30,34–37] or as hospital-based or insurance-provider-based obstetric populations [31,38]. The number of maternal deaths in our study is relatively low in comparison with the US maternal mortality ratio reported by WHO [5,6]. However, the WHO reports include all maternal deaths during pregnancy and postpartum, while our study captured only deaths occurring during delivery hospitalization.

Aging leads to non-specific deterioration of most physiological functions [41], with chronological age being a marker for (but not equivalent to) biological or reproductive age. Elevated cardiac, cerebrovascular, and respiratory morbidity in older mothers can be partly attributed to physiological changes associated with aging, including reduced cardiac reserve, muscle atrophy, atherosclerosis and other changes in the vasculature, and reduced lung function. Such changes may not be clinically apparent in the absence of pregnancy, but the added physiological burden of pregnancy can reveal a decline in organ function. This conclusion is supported by our findings that adjustment for chronic hypertension and diabetes mellitus decreased the risk of renal failure and acute cardiac morbidity among women 35 y old or older. Pre-pregnancy risk factors, including higher BMI and assisted conception, may also be partly responsible for elevated severe morbidity among older mothers; elevated risks adjusted for pre-pregnancy factors were substantially attenuated among older women. Previous obstetric history (e.g., prior cesarean delivery) and aging of reproductive organs may lead to dysfunctional labor and higher rates of obstetric interventions (e.g., cesarean delivery), which increases the risk of complications and therefore severe morbidity.

Higher rates of renal failure among older mothers are notable. Studies from North America reported a temporal increase in the rate of renal failure from 2.3 per 10,000 delivery hospitalizations in 1998–1999 to 4.5 in 2008–2009 in the US [34], and from 1.6 per 10,000 deliveries in 2003 to 2.7 in 2010 in Canada [42]. The latter study also showed that the observed increase was predominantly among women with hypertensive disorders in pregnancy, e.g., preeclampsia, while the rate of acute renal failure was 2.8 times higher among mothers ≥40 y compared with those 20–24 y. Our results indicate an even larger (16-fold) increased risk among mothers 45 y or older. However, even after additional adjustment for pre-pregnancy and pregnancy hypertension, preeclampsia, and cesarean delivery, the risk of renal failure remained significantly elevated among women ≥45 y.

Our finding of an elevated risk of sepsis (including septicemia during labor, major puerperal infection, systemic inflammatory response, and septic shock) among teen mothers is also notable. Sepsis is recognized as one of the leading causes of maternal mortality [43]. Prior studies of the association between young maternal age and sepsis are sparse. A case—control study from Scotland reported a 5-fold increase in the adjusted odds of sepsis among mothers <25 y old compared with women older than 34 y [44], suggesting an even higher risk than we observed. Untreated sepsis may lead to a cascade of sequential organ failure. It is possible that the signs of generalized infection (hypotension, tachycardia, fever, oliguria, and hypoxemia) are masked among young mothers by other compensatory mechanisms, leading to a delay in timely recognition and antibiotic treatment. Young healthy women can compensate and mask symptoms such as decreased level of consciousness and pathologic hypotension. These findings warrant further research into specific risk factors, etiopathogeneses, and compensatory mechanisms among young mothers. They also warrant increased clinical vigilance and a reduced threshold for considering early antibiotics in young mothers presenting with possible signs of infection.

An increased risk of adverse perinatal outcomes with advancing maternal age is well documented in many studies [45–55]; some have also reported elevated risks of antepartum hemorrhage, PPH, preeclampsia, and ICU admission [45,53]. However, we are aware of no previous comprehensive assessment of associations between maternal age and potentially life-threatening maternal morbidity.

Strengths of our study include a large population database collected consistently over a prolonged study period and a unique breadth of relevant information from two linked databases. These strengths enabled us to examine associations between maternal age and severe maternal morbidity due to a wide variety of specific clinical conditions. Moreover, they provided better control for confounding by maternal demographic and clinical variables than previous studies when studying associations with adverse fetal/infant outcomes. For example, we were able to adjust for maternal BMI (even though we had to impute approximately 10% of values that were missing) and assisted conception. Such adjustment is not possible in most population-based studies, which are therefore prone to residual confounding by these variables. BMI was derived from the mother’s reported height and pre-pregnancy weight; this information has been included in the latest revision of the national standard birth certificate in the US (2003) [55]. BMI information is not validated using maternal pre-pregnancy measurement of height and weight, but patterns of obesity among non-Hispanic white, non-Hispanic black, non-Hispanic Asian, and Hispanic women from the National Health and Nutrition Examination Survey are comparable to those in the birth certificate data [56]. In addition, overall patterns of pre-pregnancy obesity by state from the birth certificates generally correspond with those of all adult women from the Behavioral Risk Factor Surveillance System [55,57]. Education was self-reported information abstracted from the medical charts. We used the indicator variable “less than high school graduation” as a proxy for low socioeconomic status. Teenage mothers were naturally less likely to have graduated from high school (due to their young age), which indicates that these women were more likely to be disadvantaged with respect to their socioeconomic status.

To maintain the consistency of our data sources, we limited the study to a time period when the latest version of the US birth certificate (introduced in 2003 in Washington State) was used to collect information on pregnancy, labor, delivery, and postpartum events, while hospitals continued to use ICD-9-CM codes. Using both validated data sources increases the detection rate of maternal morbid conditions [58,59]. Moreover, any potential underreporting would bias our results towards the null, as differential coding of major life-threatening medical events based on maternal age is unlikely.

Our study also has several limitations. First, despite our large sample size, we did not have sufficient statistical power to examine the association between maternal age and maternal death or very rare severe morbidities; we therefore created larger categories of morbid conditions and a composite outcome. Second, the degree of severity of some maternal morbidity was not possible to ascertain from available ICD-9-CM codes. For example, the code for eclampsia (ICD-9-CM code 642.6) did not include superimposed eclampsia (eclampsia after chronic hypertension). A specific code for superimposed eclampsia was not available in ICD-9-CM—only a code identifying mild/severe superimposed preeclampsia/eclampsia (ICD-9-CM code 642.7). This latter condition was not included in our composite outcome of severe maternal morbidity, as it would artificially inflate rates among older women, who are more likely to have chronic hypertension (and hence superimposed preeclampsia/eclampsia). The exclusion of this condition likely led to underestimated rates and ORs of severe maternal morbidity among older women. Third, errors and omissions in diagnostic coding are inevitable in all large administrative databases. In our study, however, these should have resulted in non-differential misclassification, as coding practices are unlikely to differ by maternal age. The observed rates of severe morbidity may therefore be underestimates, and the ORs biased towards the null. Finally, we were unable to adjust for income, which tends to be higher among women who delay childbearing (because some women delay childbearing in order to advance their career and achieve financial security) [60,61]. However, we did adjust for marital status, education, race, and the type of medical insurance—all of which are highly associated with income. We assessed a relatively large number of associations, and some may be statistically significant owing to chance. We excluded multiple births as they have specific pregnancy complications (with higher rates of preterm birth and maternal/perinatal morbidity), which were beyond the scope of this paper. This may affect the generalizability of our results to multifetal pregnancies. We were unable to account for potential correlations among outcomes among women who delivered more than once during the study period, because consecutive pregnancies are not linked in the BERD dataset. This could affect the risk estimates, especially for recurrent maternal morbidities such as severe PPH.

### Conclusion

Although severe maternal morbidity is uncommon, older women experience increased risks of severe morbidity during pregnancy. Counselling of older mothers should include information on both fetal/neonatal risks and maternal risks. Health care providers should be aware of the severe morbidities that can arise among older mothers and that can impact obstetrical care delivery and resource utilization during labor, delivery, and postpartum.

## Supporting information

S1 Checklist STROBE checklist. (DOCX)

S1 Fig The association between severe maternal morbidity and maternal age. Unadjusted association between severe maternal morbidity and maternal age modelled as a restricted cubic spline (left), and estimated probability of adverse outcome based on a restricted cubic spline model of maternal age and average covariate pattern per year of maternal age, adjusted for BMI, parity, race, year of childbirth, education, smoking, marital status, fetal sex, and type of health insurance (right).
(XLSX)

S1 Table Severe maternal and neonatal morbidity ICD-9-CM codes. (DOCX)

S2 Table Comparison of women excluded from the study due to delivery outside of hospital or unmatched hospital records and women who were included in the study. (DOCX)

S3 Table Perinatal mortality and severe neonatal morbidity per 100 singleton live births, Washington State, US, 2003–2013. (DOCX)

S4 Table Adjusted association between maternal age and severe maternal morbidity among singleton births, Washington State, US, 2003–2013. (DOCX)

S5 Table Maternal age and risk differences in mortality and severe maternal and neonatal morbidity among singleton births, Washington State, US, 2003–2013. (DOCX)

## Abbreviations

AFE: amniotic fluid embolism
AOR: adjusted odds ratio
ARD: adjusted risk difference
BERD: Birth Events Record Database
BMI: body mass index
CHARS: Comprehensive Hospital Abstract Reporting System
DIC: disseminated intravascular coagulation
ICU: intensive care unit
NICU: neonatal intensive care unit
OR: odds ratio
PPH: postpartum hemorrhage
