# Supplementary material for: Maternal age and severe maternal morbidity: A population-based retrospective cohort study
Source: PLoS Med. 2017 May 30;14(5):e1002307. doi: 10.1371/journal.pmed.1002307 (PMC5448726; doi:10.1371/journal.pmed.1002307)
Supplement: S1 Checklist — (DOCX) [file pmed.1002307.s001.docx]

**Strobe checklist:**

Item 1: The title indicates the study design, abstracts summarizes what was done and what was found

Item 2: Background and rationale for the study are explained

Item3: Objectives are stated

Item 4: Key elements of the study design are stated

Item 5: Study setting is described in terms of study population setting, period and geographical location, sources of data.

Item 6: Inclusion and exclusion criteria for cohort study are described, follow up period is not really an issue, as all women delivered between at 20 weeks or later gestation

Item 7: Outcomes are clearly defined, exposure is clearly defined, confounders and effect modifiers were addressed by sensitivity analyses (see previous responses).

Item 8: Method of assessment for measured variables is given, more details on BMI and education measurements were added.

Item 9: Sources of bias are addressed, including the description of women who were not included in the study due to unlinked birth and hospitalization files.

Item 10: Study size was explained, the study included all women who gave birth in Washington state.

Item 11: Quantitative variables were explained, we used grouping for BMI using standard clinical categories, clinical defined parity categories were also created (nullipara, para 1-3, and grand multipara ≥4); splines were used to adjust for these covariates. Maternal age was categorized to 5-year intervals to describe the rates and AORs.

Item 12: Statistical methods are described, logistic regression models were used with sensitivity analyses examining the effect of adjustment for various covariates (see also previous responses). Missing values were described (imputed for BMI and shown for other covariates).

Item 13: The number of women in the study population and reasons for exclusion are described.

Item 14: Characteristics of study population are given (Table 1 and 2).

Item 15: Number of outcome events is reported.

Item 16: Unadjusted and adjusted estimates are given with 95% CIs.

Item 17: Sensitivity analyses results are reported.

Item 18: Key findings are summarized with the reference to key objectives.

Item 19: Limitations of the study are discussed

Item 20: Interpretation is not implying causality in our study, the results are described for counselling purposes and interpreted as average rates given the maternal age and other covariates.

Item 21: Generalizability is discussed.

Item 22: Source of funding and conflict of interest is stated.
