# Supplementary material for: Maternal age and severe maternal morbidity: A population-based retrospective cohort study
Source: PLoS Med. 2017 May 30;14(5):e1002307. doi: 10.1371/journal.pmed.1002307 (PMC5448726; doi:10.1371/journal.pmed.1002307)
Supplement: S1 Table — (DOCX) [file pmed.1002307.s003.docx]

S1 Table: Severe maternal and neonatal morbidity, ICD-9-CM codes.

| **Severe Maternal Morbidity** | **ICD-9-CM** | **Diagnosis** |  |  |
| --- | --- | --- | --- | --- |
| 1. **Antepartum hemorrhage/Abruption (with transfusion)** | | |  |  |
|  | 641.1 | hemorrhage from placenta previa |  |  |
|  | 641.2 | premature separation of placenta/placental abruption |  |  |
|  | 641.3 | antepartum hemorrhage associated with coagulation defects |  |  |
|  | 641.8 | other antepartum hemorrhage |  |  |
|  | 641.9 | unspecified antepartum hemorrhage |  |  |
| 1. **Respiratory morbidity/Pulmonary embolism** | | |  |  |
|  | 673 | obstetric pulmonary embolism |  |  |
| **AFE** | *673.1* | *amniotic fluid embolism* |  |  |
|  | 518.0 | pulmonary collapse |  |  |
|  | 518.4 | acute oedema |  |  |
|  | 518.5 | shock - lung |  |  |
|  | 518.8 | adult RDS |  |  |
|  | 415.0 | acute cor pulmonale |  |  |
|  | 415.1 | pulmonary embolism (septic, iatrogenic, infection, other) |  |  |
|  | 799.1 | respiratory arrest (unknown causes) |  |  |
| 1. **Thromboembolism/DVT** | |  |  |  |
|  | 444.0 | arterial embolism and thrombosis of abdominal aorta | |  |
|  | 444.1 | embolism or thrombosis of thoracic aorta | |  |
|  | 453.4 | deep venous thrombosis | |  |
|  | 671.3 | deep phlebothrombosis - antepartum | |  |
|  | 671.4 | deep phlebothrombosis - postpartum | |  |
|  | 671.5 | cerebral venous thrombosis | |  |
|  | *673.2* | *pulmonary thrombosis - clot (venous thromboembolism**)* | |  |
| 1. **Cerebrovascular/CNS morbidity** | |  |  |  |
|  | 430 | subarachnoid hemorrhage |  |  |
|  | 431 | intracerebral hemorrhage |  |  |
|  | 432 | intracranial haemorrhage |  |  |
|  | *432.0* | *nontraumatic extradural hemorrhage* |  |  |
|  | *432.1* | *subdural hemorrhage* |  |  |
|  | *432.9* | *unspecified intracranial hemorrhage* |  |  |
|  | 433 | occlusion or stenosis of pre-cerebral arteries |  |  |
|  | 434 | occlusion or stenosis of cerebral arteries |  |  |
|  | 436 | cerebral seizure, apoplexy |  |  |
|  | 437.2 | hypertensive encephalopathy |  |  |
|  | 437.6 | non-pyogenic thrombosis of intracranial venous system |  |  |
|  | 342 | hemiplegia, hemiparesis |  |  |
|  | 344 | other paralytic syndromes |  |  |
|  | 348.3 | encephalopathy |  |  |
|  | 674.0 | cerebrovascular disorders in puerperium |  |  |
| 1. **Cardiac morbidity** |  |  |  |  |
|  | 401.0 | malignant essential hypertension |  |  |
|  | 402.0 | malignant essential hypertension with kidney involvement |  |  |
|  | 403.0 | malignant hypertension |  |  |
|  | 404.0 | malignant hypertension with kidney involvement |  |  |
|  | 410 | acute myocardial infarction |  |  |
|  | 417.1 | aneurism of pulmonary artery |  |  |
|  | 423.0 | hemopericardium |  |  |
|  | 423.2 | constrictive pericarditis |  |  |
|  | 423.3 | cardiac tamponade |  |  |
|  | 427.3 | atrial fibrillation/flutter |  |  |
|  | 427.4 | ventricular fibrillation/flutter |  |  |
|  | 427.5 | cardiac arrest |  |  |
|  | 428 | heart failure |  |  |
|  | 441 | aortic aneurism and dissection |  |  |
|  | 674.5 | peripartum cardiomyopathy |  |  |
| 1. **Postpartum hemorrhage (with transfusion)** | | |  |  |
|  | 666 | postpartum hemorrhage |  |  |
|  | *666.3* | *postpartum hemorrhage with coag. defects* |  |  |
| 1. **Maternal sepsis** |  |  |  |  |
|  | 038 | septicaemia |  |  |
|  | 659.3 | septicaemia during labor |  |  |
|  | 670 | major puerperal infection |  |  |
|  | 785.5 | septic shock |  |  |
|  | 995.9 | systemic inflammatory response syndrome (SIRS) |  |  |
| 1. **Acute renal failure** |  |  |  |  |
|  | 584 | acute and subacute renal failure |  |  |
|  | 586 | renal failure, unspecified |  |  |
|  | 669.3 | other renal failure (following labor and delivery) |  |  |
| 1. **Shock** | 669.1 | obstetric shock |  |  |
| 1. **Complication of anesthesia/interventions** | | |  |  |
|  | 668.0 | pulmonary complications |  |  |
|  | 668.1 | cardiac complications |  |  |
|  | 668.2 | CNS complications |  |  |
|  | 669.4 | other complications of surgery (cardiac arrest/failure following C-section) |  |  |
|  | 995.0 | other anaphylactic shock |  |  |
|  | 995.4 | shock due to anesthesia |  |  |
|  | 997.01 | CNS complications (anoxic brain damage, cerebral hypoxia) |  |  |
|  | 997.02 | iatrogenic cerebrovascular infarction (postoperative stroke) |  |  |
|  | 997.1 | cardiac complications (during procedure) |  |  |
|  | 998.0 | postoperative shock |  |  |
|  | 999.1 | air embolism (following infusion, perfusion or transfusion) |  |  |
| Uterine rupture |  |  |  |  |
|  | 665.0 | rupture of uterus before onset of labor |  |  |
|  | 665.1 | rupture of uterus during labor |  |  |
| 1. **Procedures** | |  |  |  |
|  | 99.6 | conversion of cardiac rhythm |  |  |
|  | 35 | operations on heart (valva, septa) |  |  |
|  | 36 | operations on heart (vessels) |  |  |
|  | 37 | other operations on heart |  |  |
|  | 31.1 | temporary tracheostomy |  |  |
|  | 39 | other operations on vessels (shunts, stents, sutures, etc.) |  |  |
|  | 93.90 | non-invasive mechanical ventilation |  |  |
|  | 96.0 | non-operative intubation |  |  |
|  | 96.7 | other continuous invasive mechanical ventilation |  |  |
|  | 99.0 | transfusion (any blood products) |  |  |
|  | 68.3 | subtotal abdominal hysterectomy |  |  |
|  | 68.4 | total abdominal hysterectomy |  |  |
|  | 68.9 | other and unspecified hysterectomy |  |  |
|  | 75.8 | obstetric tamponade of uterus and vagina |  |  |
|  | 89.60-89.64 | invasive hemodynamic monitoring |  |  |
| **Other** |  |  |  |  |
| Eclampsia |  | (may not include superimposed eclampsia) |  |  |
|  | 642.6 | eclampsia |  |  |
| Acute liver failure |  |  |  |  |
|  | 570 | acute and subacute liver failure |  |  |
|  | 453.0 | Budd-Chiari syndrome |  |  |
| DIC | 286.6 | disseminated intravascular coagulation |  |  |
| Sudden death | 674.9 | sudden death of unknown cause |  | |
|  |  |  |  |  |
| **Neonatal Morbidity** | **ICD-9-CM** | **Diagnosis** |  |  |
| BPD | 770.7 | Bronchopulmonary dysplasia |  |  |
|  |  | Interstitial pulmonary fibrosis |  |  |
|  |  | Wilson-Mikity syndrome |  |  |
| NEC | 777.5 | Necrotizing enterocolitis in newborn |  |  |
| ROP | 362.2 | Retrolental fibroplasia (retinopathy of prematurity) |  |  |
| IVH | 772.13 | Intraventricular hemorrhage Grade III |  |  |
|  | 772.14 | Intraventricular hemorrhage Grade IV |  |  |
| PVL | 779.7 | Periventricular leukomalacia |  |  |
| Convulsions | 779.0 | Convulsions in newborn |  |  |
| Septicaemia | 771.81 | Septicemia (sepsis) of newborn |  |  |
| Severe trauma | 767.0 | Subdural and cerebral hemorrhage |  |  |
|  | 767.4 | Injury to spine and spinal cord |  |  |
|  | 767.11 | Epicranial subaponeurotic hemorrhage (massive), subgaleal hemorrhage |  |  |
| * Only with transfusion. | |  |  |  |
| ** Includes obstetrical blood clot embolism and deep venous thrombosis with pulmonary embolism. | | |  |  |
