# Supplementary material for: Maternal age and severe maternal morbidity: A population-based retrospective cohort study
Source: PLoS Med. 2017 May 30;14(5):e1002307. doi: 10.1371/journal.pmed.1002307 (PMC5448726; doi:10.1371/journal.pmed.1002307)
Supplement: S2 Table — (DOCX) [file pmed.1002307.s004.docx]

S2 Table: Comparison of women excluded from the study due to delivery outside of hospital or unmatched records and women who were included in the study.

| **Maternal characteristics** | **Excluded women** | **Final dataset** |
| --- | --- | --- |
|  | **N (%)** | **N (%)** |
| Maternal age (years) |  |  |
| 15-19 | 6168 (6.91) | 62904 (7.59) |
| 20-24 | 24645 (27.59) | 186537 (22.52) |
| 25-29 | 26644 (29.83) | 239319 (28.89) |
| 30-34 | 20033 (22.43) | 209936 (25.35) |
| 35-39 | 9478 (10.61) | 104985 (12.68) |
| 40-44 | 2192 (2.45) | 23180 (2.80) |
| 45-49 | 148 (0.17) | 1328 (0.16) |
| 50-59 | 17 (0.02) | 80 (0.01) |
| Race |  |  |
| non-Hispanic white | 68632 (76.83) | 593057 (71.6) |
| African-American | 5119 (5.73) | 32992 (3.98) |
| Native American | 1347 (1.51) | 16789 (2.03) |
| Hispanic | 6746 (7.55) | 97260 (11.74) |
| Other | 7089 (7.94) | 85467 (10.32) |
| missing | 392 (0.44) | 2704 (0.33) |
| Maternal education (<high school) | 1360 (1.52) | 33356 (4.03) |
| Smoking during pregnancy | 9298 (10.41) | 79558 (9.61) |
| missing | 1791 (2.01) | 7879 (0.95) |
| Not married | 22067 (24.7) | 274275 (33.11) |
| missing | 5 (0.01) | 2145 (0.26) |
| Medical insurance |  |  |
| Medicaid | 23418 (26.22) | 334833 (40.43) |
| Self-pay | 3824 (4.28) | 8102 (0.98) |
| Private | 22322 (24.99) | 431456 (52.09) |
| Other^a^ | 37200 (41.65) | 47022 (5.68) |
| missing | 2561 (2.87) | 6856 (0.83) |
| Parity |  |  |
| nullipara | 37012 (41.44) | 340381 (41.10) |
| para 1-3 | 45378 (50.80) | 436366 (52.68) |
| grand multipara (≥4 births) | 5551 (6.21) | 36696 (4.43) |
| missing | 1384 (1.55) | 14826 (1.79) |
| Body mass index |  |  |
| underweight | 3183 (3.56) | 23675 (2.86) |
| normal | 41958 (46.97) | 353212 (42.64) |
| overweight | 21370 (23.92) | 191991 (23.18) |
| obese | 16284 (18.23) | 174752 (21.1) |
| missing | 6528 (7.31) | 84639 (10.22) |
| Neonatal sex (male) | 45824 (51.30) | 425007 (51.31) |
| missing | 46 (0.05) | 28 (<0.1) |

^a^ Other medical insurance includes other government insurance, student insurance, Indian Health Care, and other programs.
