# Supplementary material for: Maternal age and severe maternal morbidity: A population-based retrospective cohort study
Source: PLoS Med. 2017 May 30;14(5):e1002307. doi: 10.1371/journal.pmed.1002307 (PMC5448726; doi:10.1371/journal.pmed.1002307)
Supplement: S3 Table — (DOCX) [file pmed.1002307.s005.docx]

S3 Table: Offspring outcomes per 100 singleton live births, Washington State, USA, 2003-2013.

| **Fetal and infant morbidity** | **Maternal age** | | | | | | |
| --- | --- | --- | --- | --- | --- | --- | --- |
|  | **15-19 years (N=62,904)** | **20-24 years (N=186,537)** | **25-29 years (N=239,319)** | **30-34 years (N=209,936)** | **35-39 years (N=104,985)** | **40-44 years (N=23,180)** | **≥ 45 years (N=1,408)** |
|  | n (%) | n (%) | n (%) | n (%) | n (%) | n (%) | n (%) |
| Fetal death^a^ | 343 (0.6) | 867 (0.5) | 1035 (0.4) | 982 (0.5) | 594 (0.6) | 182 (0.8) | 24 (1.7) |
| Neonatal death | 250 (0.4) | 466 (0.3) | 553 (0.2) | 466 (0.2) | 259 (0.3) | 87 (0.4) | 9 (0.7) |
| Perinatal death^a^ | 593 (0.9) | 1333 (0.7) | 1588 (0.7) | 1448 (0.7) | 853 (0.8) | 269 (1.2) | 33 (2.3) |
| Preterm birth <34 weeks | 1331 (2.1) | 3205 (1.7) | 3645 (1.5) | 3296 (1.6) | 1908 (1.8) | 524 (2.3) | 52 (3.8) |
| <37 weeks | 5020 (8.0) | 13371 (7.2) | 16050 (6.7) | 14288 (6.8) | 8129 (7.8) | 2056 (8.9) | 186 (13.4) |
| Apgar score at 5min ≤ 3 | 526 (0.8) | 1259 (0.7) | 1510 (0.6) | 1384 (0.7) | 731 (0.7) | 198 (0.9) | 18 (1.3) |
| SGA (<10th percentile) | 5776 (9.2) | 13915 (7.5) | 14891 (6.3) | 12268 (5.9) | 6477 (6.2) | 1652 (7.2) | 106 (7.7) |
| LGA (>90th percentile) | 3783 (6.0) | 15492 (8.3) | 24949 (10.5) | 24587 (11.8) | 12954 (12.4) | 2851 (12.4) | 147 (10.6) |
| Macrosomia (≥4500g) | 509 (0.8) | 2285 (1.2) | 3932 (1.7) | 4161 (2.0) | 2255 (2.2) | 566 (2.5) | 29 (2.1) |
| NICU admission | 3791 (6.1) | 10740 (5.8) | 13000 (5.5) | 11331 (5.4) | 6125 (5.9) | 1560 (6.8) | 135 (9.8) |
| Severe neonatal morbidity‡ | 995 (1.6) | 2540 (1.4) | 2812 (1.2) | 2334 (1.1) | 1204 (1.2) | 318 (1.4) | 25 (1.8) |
| Death/Severe neonatal morbidity^a,b^ | 1553 (2.5) | 3807 (2.0) | 4322 (1.8) | 3710 (1.8) | 2015 (1.9) | 579 (2.5) | 57 (4.1) |
| Maternal and perinatal mortality/severe morbidity | 2803 (4.5) | 6567 (3.5) | 7592 (3.2) | 6550 (3.1) | 3744 (3.6) | 1082 (4.7) | 104 (7.4) |
| ^a^ Rates per 100 total births (other rates are per 100 live births) | | | | | | | |
| ^b^ Includes respiratory distress syndrome, retinopathy of prematurity, intraventricular hemorrhage (grade 3 or more), intracranial hemorrhage, sepsis, necrotizing enterocolitis, severe birth trauma and seizures. | | | | | | | |
| SGA denotes small-for-gestational-age (<10th percentile), LGA denotes large-for-gestational-age (>10th percentile), NICU denotes neonatal intensive care unit. | | | | | | | |
