# Supplementary material for: Maternal age and severe maternal morbidity: A population-based retrospective cohort study
Source: PLoS Med. 2017 May 30;14(5):e1002307. doi: 10.1371/journal.pmed.1002307 (PMC5448726; doi:10.1371/journal.pmed.1002307)
Supplement: S4 Table — (DOCX) [file pmed.1002307.s006.docx]

S4 Table: Adjusted association between maternal age and severe maternal morbidity among singleton births, Washington State, USA, 2003-2013.

| **Maternal morbidity** | | | | **Maternal age (years)** | | | | | | | | | | |  |
| --- | --- | --- | --- | --- | --- | --- | --- | --- | --- | --- | --- | --- | --- | --- | --- |
|  | | **15-19** | | | **20-24** | |  | | **30-34** | **35-39** | | | **40-44** | **45-49** |  |
|  | | **Adjusted odds ratio (95% CI)^a^** | | | | | | | | | | | | |  |
| Antepartum hemorrhage^b^ | | 0.8 | | | 0.7 | |  | | 0.8 | 1.2 | | | 1.4 | 1.4 |  |
|  |  | (0.5-1.2) | | | (0.6-0.9) | |  | | (0.7-1.0) | (0.9-1.5) | | | (0.9-2.1) | (0.4-5.9) |  |
| Respiratory morbidity | | 1.0 | | | 1.0 | |  | | 1.0 | 1.1 | | | 1.7 |  |  |
|  | | (0.7-1.4) | | | (0.8-1.2) | |  | | (0.8-1.2) | (0.9-1.4) | | | (1.2-2.3) |  |  |
| AFE | | 1.1 | | | 1.1 | |  | | 1.2 | 2.1 | | | 6.6 |  |  |
|  | | (0.2-6.3) | | | (0.3-3.6) | |  | | (0.4-3.7) | (0.7-6.4) | | | (2.0-21.7) |  |  |
| Thromboembolism/DVT | | 0.9 | | | 0.8 | |  | | 1.0 | 1.1 | | | 1.4 |  |  |
|  | | (0.6-1.3) | | | (0.6-1.0) | |  | | (0.7-1.3) | (0.8-1.5) | | | (0.9-2.3) |  |  |
| Cerebrovascular morbidity | | 0.6 | | | 0.9 | |  | | 1.2 | 1.2 | | | 2.0 | 2.6 |  |
|  |  | (0.3-1.2) | | | (0.6-1.3) | |  | | (0.8-1.8) | (0.7-1.9) | | | (1.0-3.9) | (0.4-18.6) |  |
| All cardiac morbidity | | 0.5 | | | 0.8 | |  | | 1.1 | 1.5 | | | 2.7 |  |  |
|  | | (0.3-1.0) | | | (0.6-1.1) | |  | | (0.8-1.4) | (1.1-2.0) | | | (1.8-4.1) |  |  |
| Cardiomyopathy | | 0.7 | | | 0.9 | |  | | 1.6 | 0.9 | | | 2.8 |  |  |
|  | | (0.2-2.5) | | | (0.4-1.9) | |  | | (0.9-3.1) | (0.3-2.1) | | | (1.1-7.3) |  |  |
| Severe PPH^b^ | | 1.3 | | | 1.1 | |  | | 1.0 | 1.1 | | | 1.0 | 1.4 |  |
|  | | (1.1-1.5) | | | (1.0-1.2) | |  | | (0.9-1.1) | (1.0-1.2) | | | (0.8-1.3) | (0.6-2.9) |  |
| Maternal sepsis | | 1.7 | | | 1.3 | |  | | 0.9 | 0.8 | | | 0.9 |  |  |
|  | | (1.4-1.9) | | | (1.1-1.4) | |  | | (0.8-1.0) | (0.7-1.0) | | | (0.7-1.2) |  |  |
| Renal failure | | 1.1 | | | 0.9 | |  | | 1.0 | 1.3 | | | 1.7 | 6.7 |  |
|  | | (0.5-2.4) | | | (0.5-1.5) | |  | | (0.6-1.6) | (0.8-2.2) | | | (0.8-3.5) | (1.9-23.2) |  |
| Shock | | 1.1 | | | 0.8 | |  | | 1.5 | 2.1 | | | 2.5 |  |  |
|  | | (0.4-2.6) | | | (0.4-1.5) | |  | | (0.9-2.5) | (1.2-3.8) | | | (1.1-5.7) |  |  |
| Complications^c^ | | 0.9 | | | 0.8 | |  | | 1.1 | 1.2 | | | 1.0 | 2.5 |  |
|  | | (0.7-1.1) | | | (0.7-0.9) | |  | | (0.9-1.3) | (1.0-1.5) | | | (0.8-1.4) | (1.1-5.3) |  |
| DIC | | 0.9 | | | 0.7 | |  | | 1.2 | 1.2 | | | 1.6 |  |  |
|  | | (0.4-2.3) | | | (0.4-1.3) | |  | | (0.8-1.9) | (0.7-2.0) | | | (0.7-3.4) |  |  |
| Procedures | | 1.3 | | | 1.0 | |  | | 1.0 | 1.1 | | | 1.3 | 1.7 |  |
|  | | (1.1-1.4) | | | (0.9-1.1) | |  | | (0.9-1.1) | (1.0-1.2) | | | (1.1-1.5) | (1.1-2.6) |  |
| ICU admission | | 0.1 | | | 0.7 | |  | | 0.9 | 1.0 | | | 1.2 | 3.2 |  |
|  | | (0.8-1.6) | | | (0.6-0.9) | |  | | (0.7-1.1) | (0.8-1.3) | | | (0.8-1.7) | (1.4-7.5) |  |
| Maternal death/severe morbidity/ICU admission | | 1.3 | | | 1.0 | |  | | 1.0 | 1.3 | | | 1.6 | 2.1 |  |
|  |  | (1.2-1.3) | | | (0.9-1.0) | |  | | (1.0-1.1) | (1.2-1.4) | | | (1.5-1.8) | (1.5-3.0) |  |
| Note: categories overlap, severe maternal morbidity includes also eclampsia, liver failure (see S1 Table);  DIC denotes disseminated intravascular coagulation; AFE denotes amniotic fluid embolism. | | | | | | | | | | | | | | | |
|  | | | | | | | | | | | | | | |  |
| ^a^ relative to women aged 25-29 years; adjusted for race, marital status, body-mass-index, drug use, smoking, parity, assisted conception, low education, type of health insurance, year of childbirth and male fetus; and chronic hypertension, diabetes, induction of labor, type of delivery (spontaneous vaginal, instrumental vaginal, caesarean with trial of labor, and cesarean without trial of labor), long labor, precipitous labor, gestational diabetes, hypertension in pregnancy, chorioamnionitis. Adjusted odds ratio for antepartum haemorrhage included only pre-delivery factors. | | | | | | | | | | | | | |  |  |
| ^b^ with transfusion. | |  | | |  | |  |  | |  |  |  |  |  |  |
| ^c^ complications of anesthesia and obstetric interventions. | | | | | | | | | | |  |  |  |  |  |
