# Supplementary material for: Maternal age and severe maternal morbidity: A population-based retrospective cohort study
Source: PLoS Med. 2017 May 30;14(5):e1002307. doi: 10.1371/journal.pmed.1002307 (PMC5448726; doi:10.1371/journal.pmed.1002307)
Supplement: S5 Table — (DOCX) [file pmed.1002307.s007.docx]

S5 table: Maternal age and risk differences in mortality and severe maternal and neonatal morbidity, singleton births, Washington State, USA, 2003-2013.

| **Maternal age (years)** | **15-19** | **20-24** | **25-29** | **30-34** | **35-39** | **40-44** | **45-49** | **≥ 50** |
| --- | --- | --- | --- | --- | --- | --- | --- | --- |
|  | Risk Difference (95% Confidence Interval) | | | | | | | |
| Maternal death/severe morbidity | 0.7 | 0.1 | ref. | 0.0 | 0.4 | 0.9 | 1.9 | 6.7 |
|  | (0.5 - 0.8) | (0.1 - 0.2) |  | (-0.1 - 0.1) | (0.3 - 0.5) | (0.7 - 1.1) | (1.1 - 3.1) | (2.0 - 16.9) |
| Perinatal death/severe neonatal morbidity | 0.7 | 0.2 | ref. | 0.0 | 0.1 | 0.7 | 2.3 | 1.9 |
|  | (0.5-0.8) | (0.1 - 0.3) |  | (-0.1- 0.0) | (0.0 - 0.2) | (0.5 - 0.9) | (1.4 - 3.7) | (-0.6 - 10.3) |
| Any death/severe morbidity | 1.3 | 0.4 | ref. | -0.1 | 0.4 | 1.6 | 4.4 | 7.6 |
|  | (1.1 - 1.5) | (0.3 - 0.5) |  | (-0.2 - 0.1) | (0.3 - 0.6) | (1.3 - 1.9) | (3.0 - 6.2) | (2.1 - 19.2) |
|  | Adjusted* Risk Difference (95% Confidence Interval) | | | | | | | |
| Maternal death/severe morbidity | 0.1 | -0.1 | ref. | 0.1 | 0.5 | 0.9 | 1.6 | 6.4 |
|  | (-0.1 - 0.2) | (-0.2 - 0.0) |  | (0.0 - 0.2) | (0.4 - 0.6) | (0.7 - 1.2) | (0.7 - 2.8) | (1.7 - 18.2) |
| Perinatal death/severe neonatal morbidity | -0.1 | -0.2 | ref. | 0.2 | 0.4 | 0.8 | 2.3 | 0.8 |
|  | (-0.2 - 0.0) | (-0.2 - -0.1) |  | (0.1 - 0.3) | (0.3 - 0.5) | (0.6 - 1.1) | (1.2 - 3.7) | (-1.0 - 9.0) |
| Any death/severe morbidity | 0.0 | -0.2 | ref. | 0.2 | 0.8 | 1.7 | 4.0 | 8.1 |
|  | (-0.2 - 0.1) | (-0.3 - -01) |  | (0.1 - 0.4) | (0.6 - 1.0) | (1.4 - 2.0) | (2.4 - 5.9) | (1.9 – 21.6) |
| Adjusted for race, marital status, body-mass-index, prior adverse outcomes, smoking, parity, low education, type of health insurance, year of childbirth and male fetus. | | | | | | | | |
